# Antimicrobial Desensitization: A Review of Published Protocols

**DOI:** 10.3390/pharmacy7030112

**Published:** 2019-08-09

**Authors:** Daniel B. Chastain, Vanessa Johanna Hutzley, Jay Parekh, Jason Val G. Alegro

**Affiliations:** 1 University of Georgia College of Pharmacy, Albany, GA 31701, USA; 2 Mount Sinai Hospital, Chicago, IL 60608, USA; 3 Roosevelt University College of Pharmacy, Schaumburg, IL 60173, USA

**Keywords:** antimicrobial, β-lactam, penicillin, cephalosporin, sulfonamide, allergy, hypersensitivity, desensitization, protocol

## Abstract

Antimicrobial desensitization represents a last-line option for patients with no alternative therapies, where the benefits of this intensive process must outweigh the potential harm from drug exposure. The goal of antimicrobial desensitization procedures is to establish a temporary state of tolerance to drugs that may otherwise cause hypersensitivity reactions. While no universal antimicrobial desensitization protocols exist, this review critically analyzes previously published desensitization protocols. The purpose of this review is to provide a greater insight for clinicians and institutions to ensure desensitization procedures are efficacious while minimizing potential for patient harm. With an increasing rate of antimicrobial resistance and the critical need to preserve antimicrobial agents, desensitization may represent another option in our antimicrobial stewardship toolkit.

## 1. Introduction

Inducing a state of *drug tolerance* may be required for patients unable to tolerate a particular drug or compound where no alternative is available [1]. The ability to tolerate the inciting drug is achieved through interactions with immunoglobulin (Ig)E, but may also involve other mechanisms, such as non-IgE or pharmacologic, as well as others that are undefined (Table 1). Antimicrobial desensitization, a component of inducing drug tolerance, establishes a temporary state of drug tolerance that may otherwise cause immunoglobulin (Ig)E-mediated hypersensitivity reactions (HSRs) [2]. These procedures must be undertaken carefully due to the risk of severe adverse reactions (ADRs), such as urticaria, angioedema, gastrointestinal distress, pruritus, hypotension, wheezing, and flushing, which generally occur within one hour of drug exposure [3]. Patients may experience anywhere from mild allergic reactions to life-threatening anaphylaxis. Despite these potential risks, there may still be a need to utilize antimicrobials in patients that experience severe, immediate HSRs. For example, penicillin is the only available drug option to treat syphilis in pregnant women, and penicillin desensitization has been successfully performed in these patients [4,5]. Furthermore, antimicrobial desensitization has also been studied in non-IgE-mediated reactions. Patients with *Mycobacterium tuberculosis* and a history of delayed HSR to rifampicin, isoniazid, and ethambutol were successfully desensitized [6].

Despite publication of successful strategies, widely agreed upon protocols for antimicrobial desensitization do not yet exist, as the methods used may depend on the patient, clinician’s expertise, and institutional guidelines or policies. This article will review the indications where antimicrobial desensitization should be considered, compare different desensitization protocols of commonly used antimicrobials, and explore the use of these protocols within different clinical settings. The purpose of this review is to provide a greater insight for clinicians and institutions considering developing a standard desensitization procedure that is both efficacious and minimizes potential for patient harm.

## 2. Results

### 2.1. Indications and Contraindications

When selecting appropriate candidates for antimicrobial desensitization, the benefits of this intensive process must outweigh the potential harm from drug exposure. Patients with a documented allergy to an antimicrobial may benefit from a graded drug challenge if the reaction is unknown or questionable [9,10]. For those who indeed have a true HSR, either an alternative therapy with an unrelated structural compound and acceptable therapeutic efficacy may be administered, or desensitization can be performed to induce a temporary state of tolerability [11]. In IgE-mediated immediate HSRs, although the mechanism of desensitization is not well-described, one proposed explanation is blunting the mast cell response to the drug compound by the production of antigenic determinants with gradually increasing subtherapeutic doses. This will lead to binding of IgE to an extent that will not induce cross-linking [12,13,14]. For non-IgE-mediated immediate HSRs to antimicrobials such as sulfonamides and other non-β lactams, rapid desensitization has been described; however, mechanisms are unclear [15,16,17]. Delayed allergic reactions, on the other hand, are generally mediated by IgG or IgM, soluble antigen-antibody complexes, or T-cell activation [18]. Antigen presenting cells present antigens to T-cells, which will lead to cytokine release and local inflammation HSRs [19].

#### 2.1.1. Indications

Indications for patients who should receive drug desensitization can be categorized into those with immediate or delayed HSRs (Table 2) [20,21]. The indications for both immediate and delayed reactions are similar. With most infections, there are multiple structurally unrelated drug classes that can be effective and safe options. However, when complications of patient co-morbidities, significant drug interactions, pharmacokinetic and pharmacodynamic challenges, drug availability and cost, bacterial resistance, and allergies are considered, the armamentarium of drug classes available to use narrows. When there is no reasonable alternative to a drug the patient has a severe HSR to, desensitization is warranted. The classic example for those with immediate HSR necessitating desensitization is a pregnant woman with syphilis, who has a type 1 HSR to penicillin. The only structurally unrelated compound that can be used in this case, doxycycline, carries potential fetal risk [22]. One major difference between immediate and delayed HSR is that despite immediate HSR being inherently life-threatening, the process of desensitization is effective to quell the IgE-mediated reaction. Conversely, in delayed HSR, if the reaction is severe or life-threatening, desensitization will not be helpful and should be avoided. Desensitization should be only be performed if the delayed reaction was non-severe. Before a clinician ultimately decides to desensitize, he or she must weigh the risks and benefits of this procedure [20,21].

#### 2.1.2. Contraindications

Desensitization should be considered contraindicated in situations where the risks heavily outweigh the potential benefits. Since the primary cause of death in immediate HSR is due to respiratory failure and cardiovascular collapse, patients at high risk for either of these should not undergo a desensitization protocol [23]. Desensitization should be considered absolutely contraindicated in patients who have uncontrolled asthma or chronic obstructive pulmonary disease, those who are hemodynamically unstable, and those with poorly controlled cardiovascular disease (CVD). Receipt of β-blocker treatment, previous serious anaphylactic reaction, or chronic liver, kidney, or other diseases which may put patients at high risk for a severe reaction when undergoing desensitization should be considered as relative contraindications. A *risk vs. benefit decision* must be made when considering use in these patients [20].

In delayed HSR, desensitization should be considered a contraindication in patients who experience severe, life-threatening reactions [21]. These severe reactions primarily include the heterogenous group of severe cutaneous adverse reactions (SCARs) such as Stevens-Johnson Syndrome (SJS), toxic epidermal necrolysis (TEN), drug rash with eosinophilia and systemic symptoms (DRESS) [24,25], and acute generalized exanthematous pustulosis (AGEP). Because the mechanism of these SCARs is thought to be related to T-cell activation, a decrease in IgE binding and downstream mast cell degranulation through a gradual increase in subtherapeutic doses will not improve drug tolerance. Furthermore, patients should not receive desensitization if they had previous reactions manifesting as cutaneous or systemic vasculitis, extensive mucosal ulcers, iatrogenic autoimmune reactions, severe generalized symptoms such as fever, arthritis, systemic lymphadenopathy, severe eosinophilia, or if internal organs or hematologic cell lines were involved (e.g., hepatitis, nephritis, agranulocytosis, thrombocytopenia) [21]. Although there were two successful cases of trimethoprim-sulfamethoxazole (TMP-SMX) desensitization in patients with a history of SJS [26], the severity of the reaction and risks involved do not make this an attractive option. Except for AGEP, in which one case report has shown effective desensitization with epoetin-α, desensitization should be considered an absolute contraindication in those with a history of SCARs [27]. Desensitization should be used with caution in patients with severe renal or hepatic disorders, severe CVD, and uncontrolled autoimmune disorders [9,28].

### 2.2. Review of Antimicrobial Desensitization Protocols

Antimicrobial desensitization is performed by administering fractional aliquots of the total therapeutic dose (typically dilutions of 1:100 or 1:1000) through either oral, intravenous (IV), or subcutaneous routes [3,29]. In general, the administered dose is doubled every 15 to 60 min, until the therapeutic dose has been reached. In most cases, these protocols can be completed within hours to days. The oral route is generally safer and simpler to perform than IV routes with similar efficacy [20]. This slow titration will allow for mast cell degranulation to a small extent such that clinically significant ADRs are either mild or non-existent [30].

Antimicrobial desensitization has been best described in patients with β-lactam HSR, but available protocols differ in terms of formulation, starting dose, number of steps, and dosing frequency [31]. Example desensitization protocols using oral and IV penicillin formulations are included in Table 3 and Table 4, respectively [5,32]. However, the protocol should be selected based on the proposed mechanism for the patient’s drug intolerance. Castells and colleagues developed a standardized 12-step protocol using three IV solutions with differing drug concentrations to be completed within 6 h (Table 5) [30]. This protocol has been used to successfully desensitize patients to numerous different drugs, including some antimicrobial agents [30,33]. In select patients at high risk for HSR or those who experience symptoms during the procedure, however, a more prolonged protocol may be necessary. While standardized antimicrobial desensitization protocols have been developed, these are not appropriate for use with all antimicrobial agents (e.g., TMP-SMX) or patients. In the following sections, we will review desensitization protocols for select antimicrobial agents. 

#### 2.2.1. β-Lactam Antimicrobials

**Figure 1:**
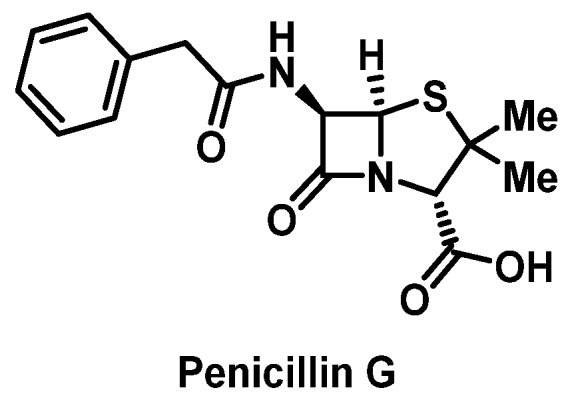
Structure of penicillin G.

**Figure 2:**
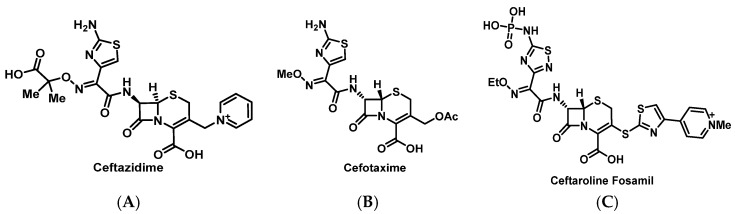
Structures of ceftazidime (**A**), cefotaxime (**B**), and ceftaroline fosamil (**C**).

The β-lactam class is associated with the highest rate of drug allergies in most epidemiological studies of ADRs [34,35]. Moreover, penicillins (Figure 1) and cephalosporins (Figure 2) are the most commonly prescribed β-lactam antimicrobials that can induce severe, life-threatening IgE-mediated HSRs [3]. To elicit an HSR, the β-lactam ring opens and binds with lysine to create the major determinant for allergic sensitivity, the penicilloyl-protein complex. Additionally, the minor determinant can occur when the β-lactam molecule undergoes isomerization to penicillanic acid, which may lead to binding with other molecules that also stimulate the immune system [3,11]. The mechanism of allergic reactions of cephalosporins, carbapenems, and monobactams may occur through mechanisms similar to those observed with penicillins; however, cross-reactivity can vary and is controversial. Historically, β-lactams were not purified, and it was thought that contamination with trace amounts of penicillins may have contributed to higher rates of cross-reactivity [36]. More recent studies show cross-reactivity rates to be much lower, but still clinically significant, with potential cross-reactivity most likely related to side chain characteristics and conformation of the β-lactam ring [37,38]. The risk is highest with oral first-generation cephalosporins, but not IV cefazolin, and with similar R-group side chains to specific penicillins [39,40,41].

##### Penicillins

Performance of penicillin skin testing (PST) has shown that approximately 90% of patients who report a penicillin allergy are not allergic. Of patients with true penicillin allergies, approximately 1% have IgE-mediated or type I reactions. Those with positive PST are at risk for an IgE-mediated allergic response to penicillin such as urticaria, angioedema, or anaphylaxis [22]. Persons who have a positive PST to one of the penicillin determinants can undergo desensitization.

Of persons reporting penicillin allergy, those with positive PST are at risk for an IgE-mediated allergic response to penicillin such as urticaria, angioedema, or anaphylaxis [22]. Persons who have a positive skin test to one of the penicillin determinants can be desensitized.

Penicillin desensitization is a relatively safe procedure that can be performed orally or IV. However, it should certainly take place in a hospital setting because severe IgE-mediated reactions can occur. Approximately one-third of patients will experience an allergic reaction during the procedure; however, these reactions tend to be mild but require prompt treatment. Modified protocols might be considered based on patient-specific symptoms, drug of choice, and route of administration. The procedure can usually be completed within 4 to 12 h, after which time the first full therapeutic dose of penicillin is administered. After desensitization, penicillin administration should be given continuously for the intended duration to maintain this temporary drug tolerance. Once the course is completed, if penicillin is required in the future, the desensitization procedure must be repeated [5,20,22,42,43]. This tolerant state is lost 24 to 36 h after discontinuation of the drug. Success rates of β-lactam desensitization has been estimated between 58 to 100%.

Many successfully utilized desensitization protocols, both oral and IV, have been published, but no large comparative studies have been performed comparing oral and IV routes of desensitization [44,45,46]. Historically, desensitization protocols started with dilutions of 10^−3^ to 10^−2^ lower than the concentration that lead to a positive PST response. Current practice suggests even lower starting doses such as a 10^−5^ to 10^−4^ dilution of the desired therapeutic concentration. These doses are then to be increased by half-log or doubling increments. The interval for IV desensitization is typically 15 min, whereas the interval recommended for oral desensitization is usually 45 to 60 min [47,48,49].

A similar approach has been adopted for patients with delayed non-life-threatening, maculopapular reactions to penicillins and has been often found to be useful in the management of patients with cystic fibrosis who have frequent requirements for IV antimicrobials and high rates of adverse antimicrobial-related reactions. In these cases, initial doses are generally higher with a variable interval between doses. Again, this procedure should be attempted only by experienced staff in the presence of full resuscitation facilities. Desensitization must not be undertaken in patients with severe cutaneous reactions with systemic features such as SJS, TENS, or DRESS [50,51].

##### Cephalosporins

Like penicillins, cephalosporins can cause immediate allergic reactions that are induced by an IgE-mediated mechanism. The manifestations are similar to those of penicillins and can occur within the first hour after administration [52,53]. Patients reporting a penicillin allergy who require treatment with a cephalosporin should undergo skin testing for both penicillin and the required cephalosporin. Results from skin testing or a single cephalosporin HSR cannot be generalized to the whole class. Patients with a confirmed penicillin allergy will require separate evaluations for each cephalosporin. If skin tests to both penicillin and cephalosporin are negative, the patient should undergo challenge with the penicillin implicated in the original reaction. If the drug challenge is negative, avoidance of any β-lactam is unnecessary. If the skin test is positive for penicillin but negative to the required cephalosporin, then the patient should be challenged with the cephalosporin [54]. Full dose challenges of oral β-lactams are warranted if there is a low probability of reaction and non-anaphylactic reaction history. Those with a history of immediate reaction should be observed for one hour, and those with delayed reactions should be observed for at least 5 days [55]. Similar to penicillin desensitization protocols, cephalosporin desensitization protocols using cefazolin [44], cefotaxime [56], ceftazidime [57], ceftriaxone [44], cefepime [44], and ceftaroline [58] have been reported (IV cephalosporin desensitization protocol is available in Supplementary Materials Table S1 in the Supplementary).

##### Carbapenems

**Figure 3:**
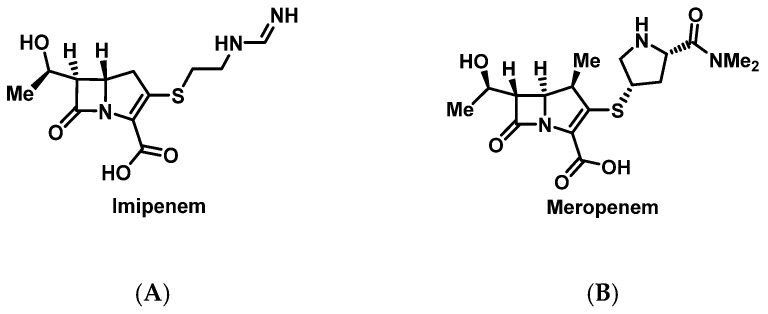
Structures of imipenem (**A**) and meropenem (**B**).

Due to a similar β-lactam ring, earlier studies suggested high rates of cross-reactivity between penicillin and carbapenems (Figure 3), such as imipenem, to be as high as 50%. However, newer prospective studies found cross-reactivity rates as low as 0.9% between penicillin and meropenem and penicillin and imipenem/cilastatin [59,60,61]. Due to stability issues, standardized protocols may not be able to be used for all carbapenems [62]. Few cases of desensitization to carbapenem have been reported, one of which increased imipenem/cilastatin concentrations by 3.3-fold every 10 min [17], while another used a standardized penicillin desensitization protocol [62].

##### Monobactams

**Figure 4:**
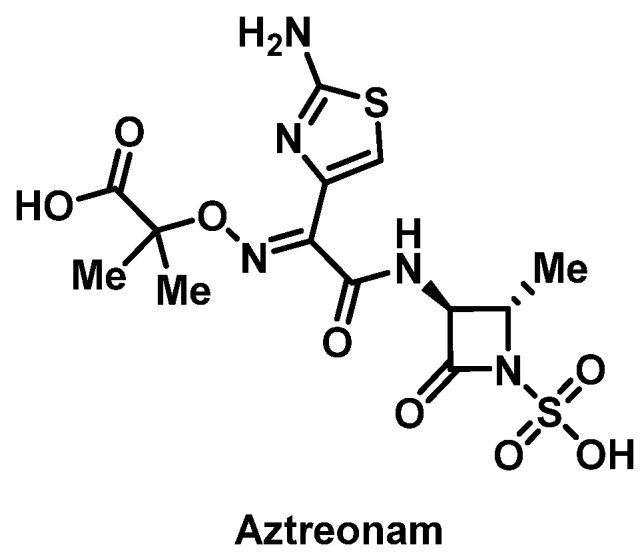
Structure of aztreonam.

Aztreonam (Figure 4) is a monobactam with a single β-lactam ring without the bicyclic ring structure characteristic of other β-lactams and is thought to be less immunogenic than penicillins or cephalosporins. Patients have been shown to tolerate aztreonam with proven immediate and delayed HSR to β-lactams [63,64,65]. Cross-reactivity between aztreonam and ceftazidime may occur due to side chain homology [66]. In a series of 11 patients with a known ceftazidime allergy, only one had a positive skin test to aztreonam, demonstrating a lower than would be predicted incidence of allergy based on molecular structure [67]. Limited data on IV aztreonam desensitization protocols are available, but protocols for inhaled aztreonam in patients with cystic fibrosis have been reported [68].

#### 2.2.2. Non-β-Lactam Antimicrobials

##### Vancomycin

**Figure 5:**
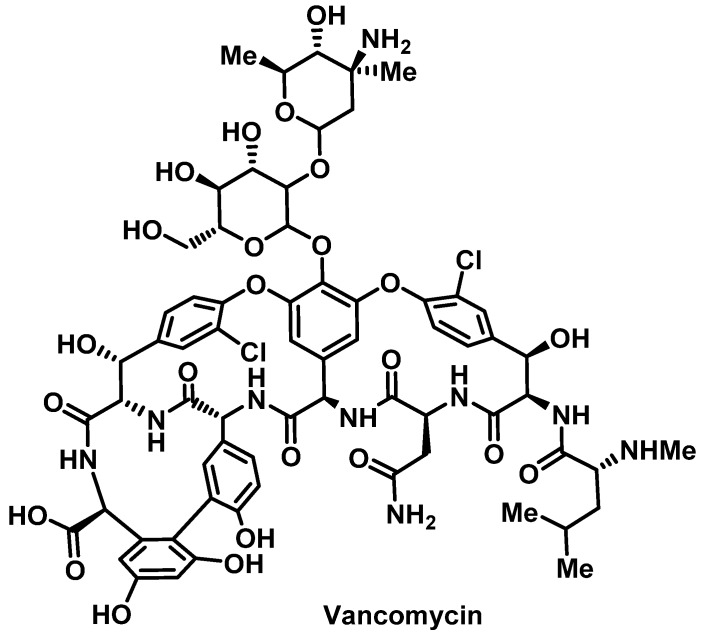
Structure of vancomycin.

Hypersensitivity reactions to vancomycin (Figure 5) include both anaphylaxis and red man syndrome (RMS), which is the most common HSR with studies reporting an incidence of 3.7–47% in infected patients and <90% in health volunteers [69,70]. Since vancomycin is also known to cause skin reactions such as erythema and pruritus, it is important to differentiate between RMS and a true allergic reaction. RMS is a pseudoallergic reaction that does not involve antibodies and results from direct stimulation of mast cells, leading to histamine release resulting in severe reactions including hypotension and muscle spasm. The incidence of RMS is dose-dependent and associated with rapid infusion of large doses [71]. Whereas 1 g of vancomycin administered IV over 30 min can often precipitate an episode, infusions of 10 mg/min rarely cause reactions, thus providing a slower infusion rate is the primary modality to prevent RMS. IgE-mediated reactions including anaphylaxis are possible with vancomycin. The potential for delayed reactions such as SJS, DRESS, and drug-induced linear immunoglobulin A-mediated bullous dermatosis, may be due to vancomycin with a severe case reported to mimic TEN [72].

Despite anaphylactic reactions to vancomycin thought to be mediated by IgE and severe RMS is mechanistically different as described above, symptomatic manifestation in patients may prove to be clinically indistinguishable. In either instance, vancomycin desensitization is recommended due to the severity of the HSR. Presently, there are no available methods to identify patients at risk for vancomycin induced HSR. Skin testing with vancomycin is likely to produce false-positive results because it directly degranulates mast cells on intracutaneous administration [73].

Vancomycin desensitization is indicated in patients with RMS that does not respond to antihistamine prophylaxis and slowing the infusion rate. It is also indicated in vancomycin induced anaphylaxis. Vancomycin desensitization attenuates mast-cell degranulation by gradually increasing serum vancomycin concentrations over several hours (rapid desensitization) to days (slow desensitization) [74]. Generally, a rapid desensitization protocol should be instituted initially, as it will enable therapeutic dosing of vancomycin within 24 h (Supplementary Materials Table S2 in the Supplementary). Slow desensitization should be reserved only for patients who fail a rapid desensitization protocol [75] (Supplementary Materials Table S3 in the Supplementary).

##### Daptomycin

**Figure 6:**
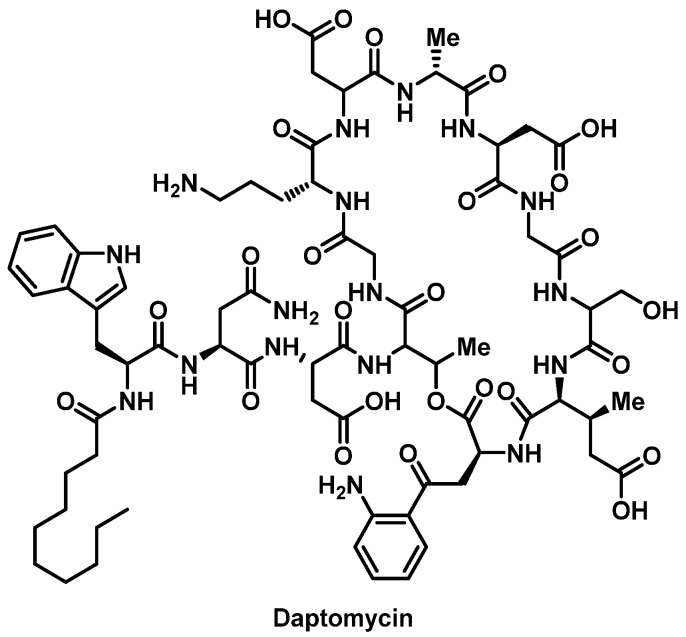
Structure of daptomycin.

Daptomycin (Figure 6) is a bactericidal lipopeptide antimicrobial used for drug-resistant Gram-positive organisms [76]. Anaphylaxis and HSRs, including eosinophilic pneumonia, AGEP, and DRESS, to daptomycin have been reported, but the mechanism remains undefined [77]. Discontinuation of daptomycin is indicated in patients with suspected anaphylaxis/HSR [78]. Limited evidence examining daptomycin desensitization is available (Supplementary Materials Table S4 in the Supplementary) [79].

##### Linezolid

**Figure 7:**
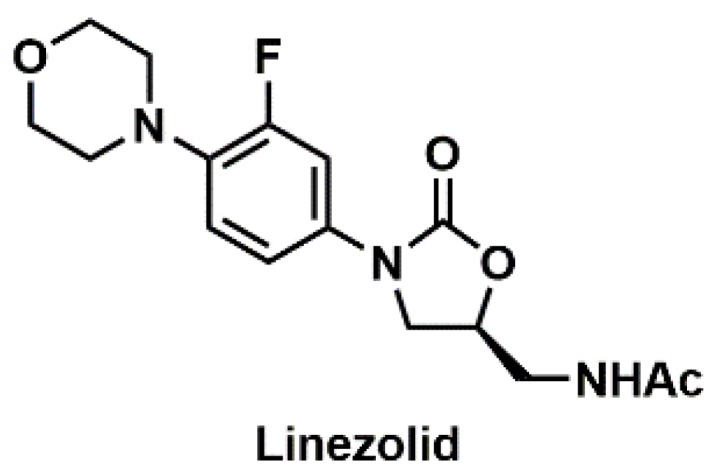
Structure of linezolid.

Linezolid (Figure 7) is an oxazolidinone with activity against Gram-positive organisms [80]. ADRs most commonly associated with linezolid include thrombocytopenia, anemia, and neutropenia. In addition, optic and peripheral neuropathy and lactic acidosis may also occur as a result of inhibition of mitochondrial protein synthesis. Immediate reactions, including hives, skin flushing, and angioedema, as well as delayed reactions, interstitial nephritis and DRESS, have been reported [81]. Although uncommon, cases of linezolid desensitization have been published [81], with one in particular administering an IV formulation of linezolid via oral route [82].

##### Clindamycin

**Figure 8:**
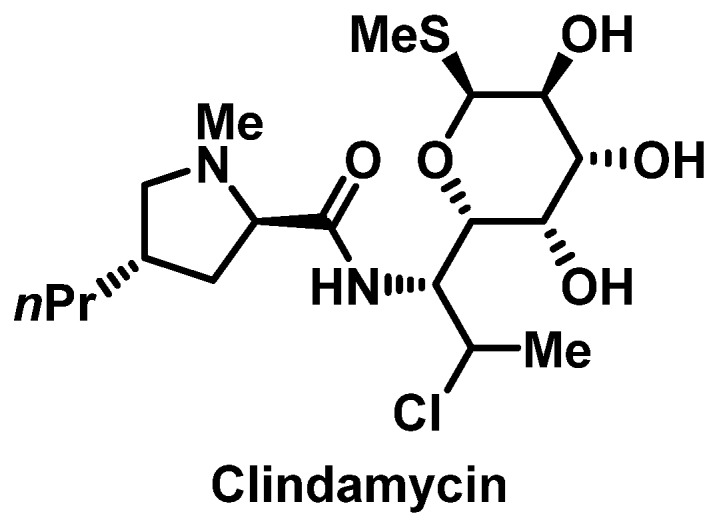
Structure of clindamycin.

Clindamycin (Figure 8) can be associated with immediate and delayed allergic reactions, though the prevalence of either is rare [83,84]. Few cases of anaphylaxis to clindamycin have been reported [85,86,87]. Though clindamycin is generally well tolerated, it can also cause severe cutaneous ADRs, such as AGEP or TEN [83,88,89,90]. While rare, clindamycin desensitization has been reported in the literature (Supplementary Materials Table S5 in the Supplementary) [91,92].

##### Macrolides

**Figure 9:**
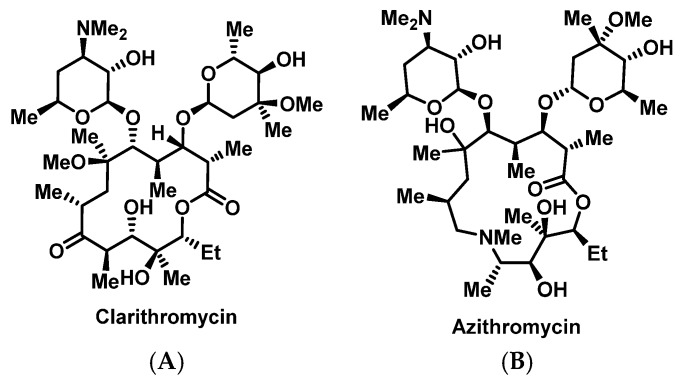
Structures of clarithromycin (**A**) and azithromycin (**B**).

Macrolides are commonly used antimicrobials, especially in community settings, and are classified based on the number of carbon atoms in their chemical structure (Figure 9) [84]. The main structural component of macrolides is the lactone ring, and based on the number of atoms in this ring macrolides can be subdivided into four classes [93]. Macrolides have variable cross-reactivity with other macrolides; however, it has not been studied thoroughly [94,95,96]. In several small case studies, cross-reactivity was described involving erythromycin and azithromycin [95,96]. Urticarial, angioedema, anaphylaxis, SJS, and even TEN are potential allergic reactions associated with the macrolides [29]. These allergic reactions are uncommon and range between 0.4–3% [97]. Additionally, a macrolide-specific IgE HSR has not been reported [98]. However, a study was able to detect drug-specific IgE antibodies in a patient experiencing an allergic reaction to erythromycin [99]. As a result, successful desensitization to azithromycin [98] and clarithromycin [100] have been reported (Supplementary Materials Table S6 in the Supplementary). Failed desensitization to a single macrolide does not predict failure to other agents in this drug class, and additional desensitization should be considered.

##### Aminoglycosides

**Figure 10:**
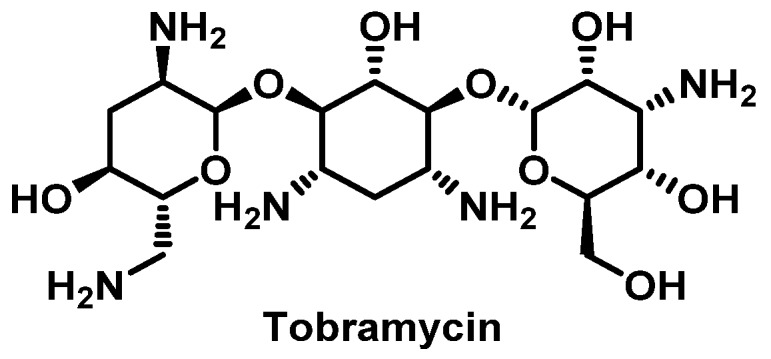
Structure of tobramycin.

Aminoglycosides can be subdivided into 2 classes: The streptidine group, which includes streptomycin, and the desoxystreptamine group, which includes kanamycin, amikacin, gentamicin, tobramycin (Figure 10), and neomycin [90]. Aminoglycosides can rarely cause both immediate and nonimmediate HSR; however, cross-reactivity is common, approaching 50% or more in the desoxystreptamine group [101]. Contact dermatitis is the most frequent ADR associated with this class of antimicrobials, specifically topical aminoglycosides [102]. Other cutaneous manifestations like urticaria, maculopapular rash, fixed drug eruption and TEN have been reported [103,104]. Anaphylaxis to aminoglycosides is very uncommon [105,106,107]. Desensitization is possible for IV (Supplementary Materials Table S7 in the Supplementary) and inhaled tobramycin, as well as those with urticaria or angioedema due to streptomycin [108,109,110].

##### Tetracyclines

**Figure 11:**
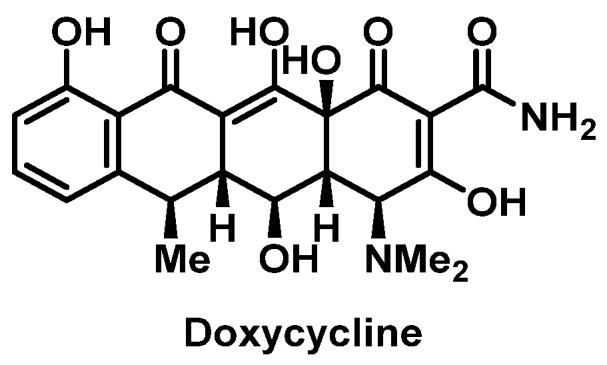
Structure of doxycycline.

Few cases of IgE-mediated reactions to tetracycline [111,112], minocycline [113,114], and doxycycline (Figure 11) [115] have been reported. It remains unknown if these HSR were drug or drug class specific since tetracyclines share a similar core structure but have different side chains. Doxycycline desensitization has been in two separate patient cases [116,117]. Due to limitations with the solubility of low doses of oral doxycycline, slow IV pushes were administered every 15 min for 4 h (cumulative dose of 98.888 mg). This was followed by an oral dose of 100 mg and subsequent initiation of 100 mg twice daily, which was reportedly well-tolerated in both patients.

##### Sulfonamides

Sulfonamide drug eruptions were the earliest described antimicrobial HSRs, with recent health plan data showing 4.3% of patients reporting a sulfonamide allergy. The most serious form of HSR to antimicrobials containing sulfonamide pharmacophores are delayed T-cell mediated reactions, such as SJS, DRESS, and TEN [55].

**Figure 12:**
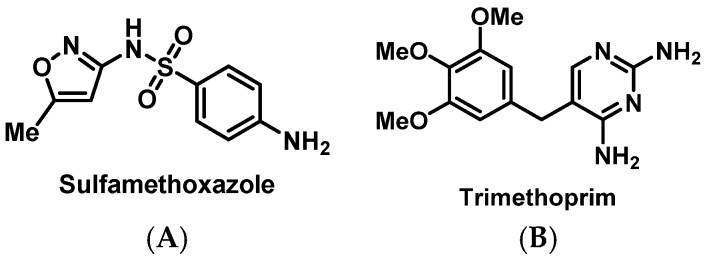
Structures of sulfamethoxazole (**A**) and trimethoprim (**B**).

SMX-TMP (Figure 12) desensitization protocols have been used with high success in patients with sulfonamide ADRs at outpatient clinics in order to treat infections and to provide prophylaxis and treatment for *Pneumocystis jirovecii* pneumonia (PJP) [118]. In one study, mild symptoms of urticaria (13%) and rashes (54%) were reported during the procedure, and desensitization was discontinued in 11% of patients due to intolerance [119].

Desensitization is primarily indicated for patients who exhibit only non-life-threatening immediate reactions, such as fevers, rashes, swelling, and urticaria. For serious delayed reactions (specifically SJS), although there have been reports of successful desensitization in these patients, it is not recommended by most clinicians because of the exceptionally high risk of a fatal ADR [120]. Therefore, the desensitization protocols reviewed here are for patients with either immediate HSR or those with non-severe delayed HSR.

Desensitization has been successfully performed in the outpatient setting; however, it is imperative that nurses, physicians, and other experts in anaphylaxis should be readily available during the procedure. The desensitization procedure has best been described using the oral route. The duration of treatment will vary greatly depending on the severity of drug intolerance and patient risk. Complete SMX-TMP can occur in just 7 h, with patients starting at a SMX-TMP dose of 4 mg/0.8 mg, with subsequent dose increases every hour until the target dose of 400 mg/80 mg SMX-TMP is reached (Supplementary Materials Table S8 in the Supplementary) [121]. Alternatively, desensitization can be conducted using the oral route over a 10-day period to reach the final dose of 1600 mg/320 mg SMX-TMP [122] (Supplementary Materials Table S9 in the Supplementary). Each of the doses during the first nine days was administered every 30 min, while the two doses on the final day were separated by 3 h.

Clinicians will have to determine which protocol is best suited for their patients. If patients have minor ADRs to SMX-TMP and are otherwise healthy, a rapid 7-h desensitization procedure may be used. In more at-risk patients, it is crucial to desensitize using a slower, safer procedure to minimize patient risk.

##### Metronidazole

**Figure 13:**
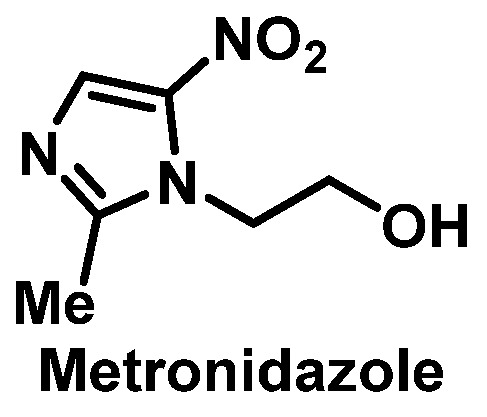
Structure of metronidazole.

A variety of HSR to metronidazole (Figure 13) have been reported, including fixed drug eruption [123], serum sickness [124], SJS [125], and anaphylaxis [126]. A potential for cross-reactivity exists between metronidazole and other nitroimidazoles (e.g., tinidazole) [127]. In patients at high risk of immediate HSR to metronidazole without alternatives, such as in the case of *Trichomonas vaginalis*, desensitization should be performed [22]. Multiple case series have been published confirming the efficacy of both IV (Supplementary Materials Table S10 in the Supplementary) and oral (Supplementary Materials Table S11 in the Supplementary) desensitization protocols [128,129,130].

##### Fluoroquinolones

**Figure 14:**
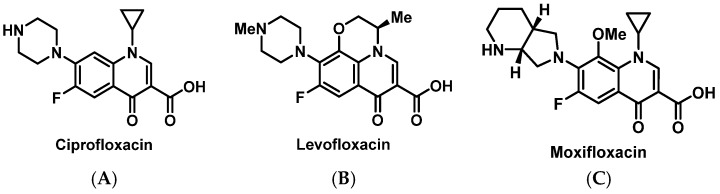
Structures of ciprofloxacin (**A**), levofloxacin (**B**), and moxifloxacin (**C**).

Fluoroquinolones (FQs) (Figure 14) may lead to delayed- and immediate-type HSR, which can include urticaria, angioedema, hypotension, and even anaphylaxis. IgE-mediated anaphylaxis to FQs appears to be increasing to a rate comparable to β-lactams [131]. Mast cell surface receptor Mas-related G protein-coupled receptor X2 (MRGPRX2) activation has been suggested as a potential mechanism for FQ-induced HSRs [132]. Of 55 patients who reported a history of immediate-type HSR to FQs which occurred within the last 4 years, 55% had detectable FQ-specific IgE antibodies determined by radioimmunoassay [133]. Higher radioimmunoassay results were found in patients with HSRs occurring within the last 8 months, which may suggest that FQ-specific IgE antibodies wane over time. In addition, cross-reactivity between FQs is likely due to similarities in core structure, as confirmed by detecting IgEs against more than one FQ. Successful desensitization to ciprofloxacin (Supplementary Materials Table S12 in the Supplementary) [134,135,136], levofloxacin [137], and moxifloxacin [138] has been reported in a variety of patients with a history of immediate-type HSRs.

#### 2.2.3. Antifungals

Tolerability of antifungals continues to be problematic. Although antifungals may cause a variety of ADRs, few cases of immediate HSRs have been reported [139]. With an increasing number of immunocompromised patients at risk of invasive fungal infections, management of antifungal associated ADRs is critical, as alternative therapies are limited or potentially nonexistent.

##### Polyene Antifungals

**Figure 15:**
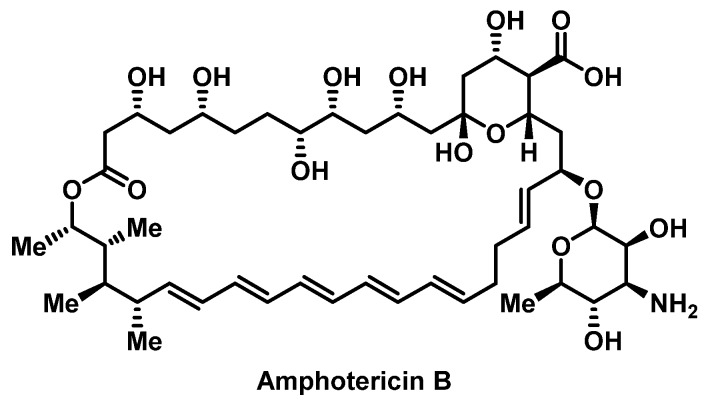
Structure of amphotericin B.

Amphotericin B (Figure 15) is a polyene antifungal with the broadest spectrum of activity compared to other currently available antifungals. Lipid associated formulations of amphotericin B (LFAB), which includes amphotericin B lipid complex (ABLC), liposomal amphotericin B (LAmB) and amphotericin B colloidal dispersion (ABCD), were introduced to mitigate the toxicities associated with amphotericin B deoxycholate (AmBD), infusion-related ADRs and nephrotoxicity [140]. The rate of anaphylaxis to LFAB has been reported to be 1.4%, but limited data on available on immediate-type HSRs since most studies involving AmBD and LFAB focus on treatment efficacy and the previously mentioned ADRs [141]. In addition, the mechanism of LFAB associated anaphylaxis is poorly understood. A case series that included 4 children with immediate-type 1 HSRs to LAmB reported the use of a 7-step protocol which resulted in successful desensitization in all patients (Supplementary Materials Table S13 in the Supplementary) [142].

##### Triazole Antifungals

**Figure 16:**
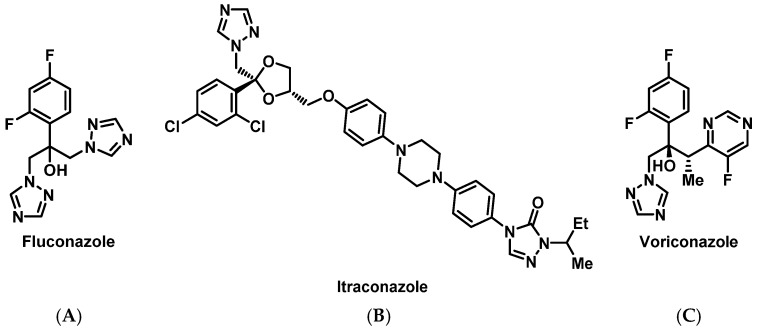
Structures of fluconazole (**A**), itraconazole (**B**), and voriconazole (**C**).

Triazole antifungals represent the mainstay of treatment for invasive fungal infections. While ADRs with triazole antifungals (fluconazole (Figure 16A), itraconazole (Figure 16B), voriconazole (Figure 16C), posaconazole, and isavuconazole) are frequently reported, HSRs including angioedema and anaphylaxis are rare [143]. In select situations (e.g., candidiasis), switching to another triazole antifungal may represent a reasonable approach to managing triazole-related HSRs. However, cross-reactivity between triazole antifungals varies with some patients tolerating alternative triazoles, whereas others experienced recurrent HSRs [143]. Voriconazole has been used successfully in patients experiencing HSRs to fluconazole and itraconazole [144,145], while isavuconazole has been used successfully in a patient developing angioedema to voriconazole [143]. Alternatively, desensitization may represent the only therapeutic option. Desensitization to fluconazole has been successfully performed using a rapid protocol in HIV-uninfected patients (Supplementary Materials Table S14 in the Supplementary) [146] and protocol spanning several-days in HIV-infected patients (Supplementary Materials Table S15 in the Supplementary) [146,147]. Additional reports have described successful desensitization to itraconazole capsules (Supplementary Materials Table S16 in the Supplementary) [148,149] and suspension [149], as well as to voriconazole (Supplementary Materials Table S17 in the Supplementary) [150].

##### Echinocandins

**Figure 17:**
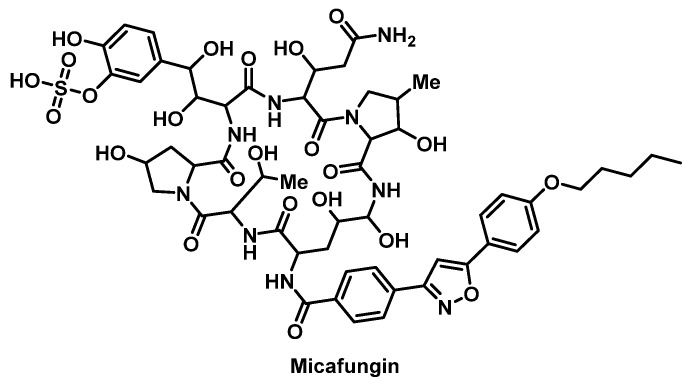
Structure of micafungin.

Although rare, delayed-type HSRs, including maculopapular rashes, erythema multiforme, and SJS, to echinocandins (caspofungin, micafungin (Figure 17), anidulafungin) occur more often than immediate-type with anaphylaxis occurring in approximately 0.2% of patients [150]. There has only been one report of echinocandin desensitization which used a 12-step protocol that began with an initial dose of 0.003 mg that was doubled with every dose administered at 15-min intervals [151]. A final dose of 150 mg was administered over 186 min.

#### 2.2.4. Antivirals

Antivirals and antiretrovirals may cause both immediate and delayed HSR, including rash, angioedema, constitutional symptoms (e.g., fever, chills, myalgias) and severe cutaneous reactions like SJS and TEN [152]. Proposed mechanisms of HSR to antivirals include the hapten hypothesis, the pharmacologic interaction (PI) model, and the altered peptide repertoire model [153]. In the hapten hypothesis, the drug or its metabolites create a neo-antigen by covalently bonding to self-proteins [154]. The PI model postulates that the antiviral stimulates T-cell activation by direct binding to human leukocyte antigen (HLA) alleles and/or T-cell receptors [155,156]. In the altered peptide repertoire model, it is suggested that the drug occupies the peptide binding cleft of the HLA molecule, which leads to alteration of the self-peptide repertoire, and thus an altered T-cell response. This altered self-repertoire has been described specifically with HLA-B*57:01 mediated abacavir hypersensitivity [157]. Those with human immunodeficiency virus (HIV) infection have an overall higher risk of HSR compared to HIV-uninfected patients, especially those with lower CD4+ T-cell counts [158]. The concern for alternative antiviral toxicity or the presence of drug resistance or major drug interactions with antiretrovirals may warrant desensitization.

**Figure 18:**
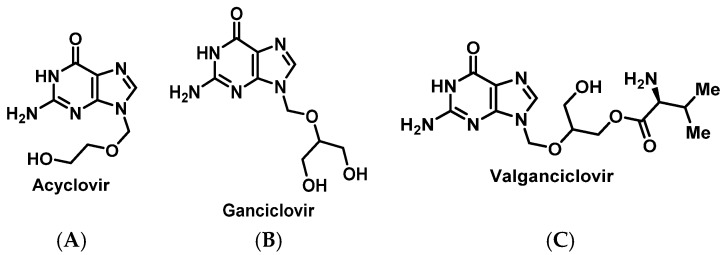
Structures of acyclovir (**A**), ganciclovir (**B**), and valganciclovir (**C**).

For antivirals targeting herpesviruses, alternative therapy if a patient experiences an HSR remains limited to toxic agents such as foscarnet and cidofovir. Fortunately, desensitization protocols have been described for acyclovir (Figure 18A, Table S18 in the Supplementary) and more recently for valganciclovir (Figure 18B,C, Table S19 in the Supplementary) [159,160,161,162,163,164].

**Figure 19:**
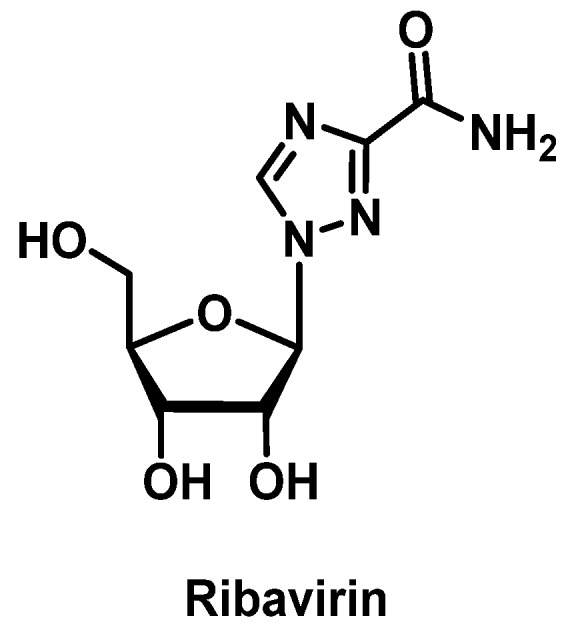
Structure of ribavirin.

**Figure 20:**
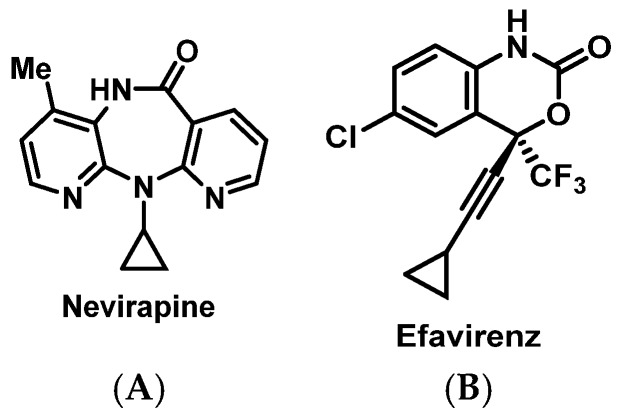
Structures of nevirapine (**A**) and efavirenz (**B**).

In contrast, with therapeutic advancements in the treatment of hepatitis C virus (HCV) and HIV, there are a variety of safe and effective alternatives if a patient has a history of HSR. However, ribavirin (Figure 19) may still be used in certain HCV genotypes depending on patient’s stage of cirrhosis and previous treatment experience [165]. Ribavirin desensitization has been described to occur over a period of 4 to 5 weeks to achieve therapeutic doses of 800 to 1000 mg daily [166,167]. In addition, desensitization protocols have been described for many antiretrovirals (ARVs), including nevirapine (Figure 20A) [168,169], efavirenz (Figure 20B) [170], nelfinavir [171,172], darunavir [173], zidovudine [174], and enfuvirtide [152,175,176,177]. However, one of the most notorious ARV associated with an HSR is abacavir. However, given the potentially fatal nature of these reactions via the mechanism described above, genetic testing for HLA-B*57:01 should be done prior to starting abacavir, and re-challenge or desensitization should never be done in patients with any history of HSR to this drug [178].

## 3. Discussion

Before performing a desensitization procedure, the patient must be in stable condition and necessary clinicians should be present. The protocol should be overseen by a physician with capabilities to treat anaphylaxis, including emergent intubation if respiratory collapse occurs. Nurses should be trained to recognize early signs of anaphylactic shock, and other allergists and intensivists should be immediately available for consultation. Antimicrobial desensitization is generally first performed in an inpatient setting, and upon successful treatment, subsequent desensitization procedures can occur at an outpatient clinic.

Patient-specific factors, type of HSR, drug, route of administration, and clinician experience affect the likelihood of successful desensitization [20]. Breakthrough symptoms are dose-dependent and often occur later in the protocol in ≤20% of patients, however immediate management is required, and the protocol must be stopped. More than 90% of reactions will resolve spontaneously. Although if symptoms persist or worsen, treatment is indicated. One or more of the following should be administered based on symptomology: Antihistamines for pruritis or urticaria, inhaled bronchodilators for shortness of breath, or epinephrine for hypotension or laryngeal edema. The protocol can be resumed by repeating the dose that caused the reaction or by restarting at a lower dose unless the patient experienced a potentially fatal reaction (e.g., serum sickness, laryngeal edema that does not respond to epinephrine), whereby the protocol should be discontinued. While considered high-risk, no fatal outcomes have been reported when desensitization protocols have been followed.

Patients should be informed to continue reporting an allergy to the particular drug as tolerance induced by desensitization reverses within hours or days in the absence of the drug [20,179]. If future treatments are required, desensitization should be repeated, or daily administration of the drug may result in ongoing tolerance [179].

## 4. Materials and Methods

A systematic literature search using PubMed, MEDLINE, and Google Scholar databases was performed. Search terms included antibiotic, antimicrobial, hypersensitivity, allergy, skin test, desensitization, indications, contraindications, β-lactam, penicillin, cephalosporin, carbapenem, monobactam, vancomycin, daptomycin, clindamycin, macrolide, aminoglycoside, fluoroquinolone sulfonamide, tetracycline, antiviral, antifungal, antiretroviral, and protocol. Articles were screened by title and abstract for possible inclusion, and references within articles of interest were scanned to capture additional sources.

## 5. Conclusions

A common problem encountered with the rise in antimicrobial resistance is that therapeutic options are becoming increasingly limited and, in some situations, toxic. In addition, if a patient experiences a severe HSR to one of the remaining antimicrobials, then alternative options may be limited, and desensitization must be considered. Desensitization may be used in patients with severe immediate HSR or non-severe delayed HSR when no other therapeutic alternative is available, but should be avoided in those with immediate-type HSR at high risk for respiratory or cardiovascular collapse, and those with delayed hypersensitivity with severe reactions such as SJS, TEN, DRESS, or internal organ involvement. No universal antimicrobial desensitization protocols exist; however, this review summarizes numerous cases and case series of desensitization protocols that have been successful in allowing patients to receive therapeutic doses of the antimicrobial that previously caused an HSR. Although there is significant heterogeneity among the different protocols described in this review, there are several common themes worth noting: (1) intervals between doses were generally 15–30 min, with a longer interval between the final dose, which allowed for adequate observation time to determine if the patient experienced a reaction (2) for patients at high risk of an ADR to desensitization or those who did not tolerate a rapid desensitization protocol, performing a slower desensitization over several days or pre-medicating with antihistamines or corticosteroids, was generally successful (3) the overall process is very time-intensive, with the majority of protocols taking several hours and some up to several weeks, and (4) if done in a controlled and systematic manner, desensitization is a safe and effective therapeutic modality to ensure administration of a necessary drug.

Currently, desensitization is a last-line option for patients who have no other alternative. With a better understanding of safe desensitization practices, could there be a paradigm shift towards desensitizing a patient to use a first-line option, even if alternatives exist? For instance, in a patient with MSSA endocarditis with anaphylaxis to a β-lactam, vancomycin could be started initially for treatment, while concomitant desensitization to nafcillin be performed, which would allow the patient to complete six weeks of optimal therapy with nafcillin. This can also be a way to avoid full courses of antimicrobials with significant, potentially permanent toxicities such as FQs, aminoglycosides, or polymyxins. Additionally, most protocols described in this review involve patients who experienced severe immediate-type HSRs (e.g., urticaria, angioedema, anaphylaxis) or non-severe delayed-type HSRs (e.g., rash), however studies involving patients with severe delayed-type HSR are lacking. Though there have been a few reports of successful desensitization in patients with severe delayed-type HSRs, the risk of potentially fatal outcomes outweighs the benefit of drug administration. An important area of study would be to investigate how drugs that have previously caused severe cutaneous ADRs such as SJS or DRESS can be safely administered to patients who need them. Given the current state of drug resistance and the need to preserve the antimicrobials we have currently, desensitization may become another option in our antimicrobial stewardship toolkit to optimize antimicrobial use.

## Figures and Tables

**Table 1:** Characteristics of drug intolerance protocols.

Underlying Mechanism	Initial Dose	Protocol Duration	Potential Outcome	Duration of Induced Tolerance	Examples
Immunologic IgE (desensitization)	Micrograms	Hours	Blunting the mast cell response	Temporary	β-lactam antibiotics
Immunologic non-IgE	Milligrams	Hours to days (e.g., 6 h to 10 days)	Unknown	Temporary	Delayed cutaneous reactions to SMX-TMP in HIV-infected patients
Pharmacologic	Milligrams	Hours to days (e.g., 2 h to 5 days)	Inhibition of tyrosine kinases and STAT6 resulting in IL-4 suppression	Temporary	Aspirin-exacerbated respiratory disease (AERD)
Undefined	Micrograms to milligrams	Prolonged; days to weeks	Unknown	Temporary	Allopurinol-induced pruritic maculopapular rash

Data adapted from [1,7,8,9]. IL, interleukin; STAT6, signal transducer and activator of transcription 6 signaling pathway; SMX-TMP, sulfamethoxazole-trimethoprim.

**Table 2:** Indications and Contraindications for Antimicrobial Desensitization [20,21,23].

	Indications	Relative Contraindications	Absolute Contraindications
**Immediate HSR**	No safe and effective alternative is available Benefits > risks	Receiving β-blockers Previous severe anaphylactic reaction Chronic hepatic or renal disease	Severe asthma or COPD Hemodynamic instability Uncontrolled CVD
**Delayed HSR**	No safe and effective alternative is available Previous delayed drug reaction was not severe Benefits > risks	AGEP Chronic hepatic or renal disease Chronic severe cardiac disease Uncontrolled autoimmune disorders	SJS TEN DRESS Cutaneous/systemic vasculitis Extensive mucosal ulcers Autoimmune drug reactions Internal organ involvement Cytopenias

AGEP, acute generalized exanthematous pustulosis; COPD, chronic obstructive pulmonary disease; CVD, cardiovascular disease; DRESS, drug rash with eosinophilia and systemic symptoms; HSR, hypersensitivity reaction; SJS, Stevens-Johnson Syndrome; TEN, toxic epidermal necrolysis.

**Table 3:** Oral penicillin suspension (**A**) and tablet (**B**) desensitization protocols.

**A**				
**Dose Number**	**Penicillin Concentration (Units/mL)**	**Amount (mL)**	**Dose (Units)**	**Cumulative Dose (Units)**
1	1000	0.1	100	100
2	1000	0.2	200	300
3	1000	0.4	400	700
4	1000	0.8	800	1500
5	1000	1.6	1600	3100
6	1000	3.2	3200	6300
7	1000	6.4	6400	12,700
8	10,000	1.2	12,000	24,700
9	10,000	2.4	24,000	48,700
10	10,000	4.8	48,000	96,700
11	80,000	1.0	80,000	176,700
12	80,000	2.0	160,000	336,700
13	80,000	4.0	320,000	656,700
14	80,000	8.0	640,000	1,296,700
*Interval between doses was 15–30 min, with a total time of 4–8 h. Observation before full parenteral therapeutic dose was 30 min. Each dose was diluted in 30 mL of water prior to oral administration.*				
**B**				
**Dose Number**	**Penicillin Concentration (mg/mL)**	**Amount (mL)**	**Dose (mg)**	**Cumulative Dose (mg)**
1	0.5	0.1	0.05	0.05
2	0.5	0.2	0.1	0.15
3	0.5	0.4	0.2	0.35
4	0.5	0.8	0.4	0.75
5	0.5	1.6	0.8	1.55
6	0.5	3.2	1.6	3.15
7	0.5	6.4	3.2	6.35
8	5.0	1.2	6.0	12.35
9	5.0	2.4	12.0	24.35
10	5.0	5.0	25.0	49.35
11	50	1.0	50.0	100.0
12	50	2.0	100.0	200.0
13	50	4.0	200.0	400.0
14	50	8.0	400.0	800.0

Data adapted from [5,32].

**Table 4:** Intravenous penicillin desensitization protocol.

Dose Number	Penicillin Concentration (mg/mL)	Infusion Rate (mL/h)	Dose (mg)	Cumulative Dose (mg)
1	0.01	6	0.015	0.015
2	0.01	12	0.03	0.045
3	0.01	24	0.06	0.105
4	0.01	50	0.125	0.23
5	0.1	10	0.25	0.48
6	0.1	20	0.5	1.0
7	0.1	40	1.0	2.0
8	0.1	80	2.0	4.0
9	0.1	160	4.0	8.0
10	10.0	3	7.5	15.0
11	10.0	6	15.0	30.0
12	10.0	12	30.0	60.0
13	10.0	25	62.5	123.0
14	10.0	50	125.0	250.0
15	10.0	100	250.0	500.0
16	10.0	200	500.0	1000.0

Data adapted from [32]. Intravenous administration was given via continuous infusion pump Interval between doses was 15 min, with a total time of 4–8 h. Observation before the full therapeutic dose is 30 min.

**Table 5:** An example of the 12-step desensitization protocol using a final dose of 1000 mg.

**A**						
**Solution:**		**Total Volume**		**Concentration**		**Dose**
Solution 1		100 mL		0.100 mg/mL		10 mg
Solution 2		100 mL		1.00 mg/mL		100 mg
Solution 3		100 mL		10.00 mg/mL		1000 mg
**B**						
**Step**	**Solution#**	**Rate (mL/hr)**	**Time (minutes)**	**Volume (mL)**	**Dose (mg)**	**Cumulative dose (mg)**
1	1	2	15	0.5	0.050	0.050
2	1	5	15	1.25	0.125	0.175
3	1	10	15	2.5	0.25	0.425
4	1	20	15	5	0.5	0.925
5	2	5	15	1.25	1.25	2.175
6	2	10	15	2.5	2.5	4.675
7	2	20	15	5	5	9.675
8	2	40	15	10	10	19.675
9	3	10	15	2.5	25	44.675
10	3	20	15	5	50	94.675
11	3	40	15	10	100	194.675
12	3	80	60.40	80.53	805.325	1000

Data adapted from [30,33].

## Supplementary Materials

The following are available online at https://www.mdpi.com/2226-4787/7/3/112/s1, Table S1. Intravenous cephalosporin desensitization protocol. Table S2. Rapid intravenous vancomycin desensitization protocol. Table S3. Slow intravenous vancomycin desensitization protocol. Table S4. Daptomycin desensitization protocol. Table S5. Oral clindamycin desensitization protocol. Table S6. Oral clarithromycin desensitization protocol. Table S7. Intravenous tobramycin desensitization protocol. Table S8. Rapid Oral Sulfamethoxazole-Trimethoprim (SMX-TMP) Desensitization Protocol. Table S9. Slow Oral Sulfamethoxazole-Trimethoprim (SMX-TMP) Desensitization Protocol. Table S10. Intravenous metronidazole desensitization protocol. Table S11. Oral metronidazole desensitization protocol. Table S12. Intravenous ciprofloxacin desensitization protocol. Table S13. Intravenous liposomal amphotericin B (LAmB) desensitization protocol. Table S14. Rapid oral fluconazole desensitization protocol. Table S15. Several-day oral fluconazole desensitization protocol. Table S16. Oral itraconazole capsule desensitization protocol. Table S17. Intravenous voriconazole desensitization protocol. Table S18. Oral acyclovir desensitization protocol. Table S19. Oral valganciclovir desensitization protocol.
