# Supplementary material for: Antimicrobial Desensitization: A Review of Published Protocols
Source: Pharmacy (Basel). 2019 Aug 9;7(3):112. doi: 10.3390/pharmacy7030112 (PMC6789802; doi:10.3390/pharmacy7030112)
Supplement: Supplementary file 1 [file pharmacy-07-00112-s001.pdf]

Title: Antimicrobial Desensitization: A Review of Published Protocols

Authors: Daniel B. Chastain[1], Vanessa Johanna Hutzley[2], Jay Parekh[3], Jason Val G. Alegro[2,3*]

1. University of Georgia College of Pharmacy, Albany, GA 31701, USA; daniel.chastain@uga.edu
2. Mount Sinai Hospital, Chicago, IL 60608 USA
3. Roosevelt University College of Pharmacy, Schaumburg, IL 60173, USA

* Correspondence: jalegro@roosevelt.edu; Tel.: 1-847-619-7290

Table S1. Intravenous cephalosporin desensitization protocol [1].

**Dose Number**	**Goal Therapeutic Dose – 1 gram IV cephalosporin**		**Goal Therapeutic Dose – 2 gram IV cephalosporin**	
	**Dose (mg)**	**Cumulative Dose (mg)**	**Dose (mg)**	**Cumulative dose (mg)**
**1**	0.1	0.1	0.1	0.1
**2**	0.2	0.3	0.4	0.5
**3**	1.0	1.3	1.0	1.5
**4**	2.0	3.3	4.0	5.5
**5**	10.0	13.3	10.0	15.5
**6**	20.0	33.3	40.0	55.5
**7**	70.0	103.3	140.0	195.5
**8**	200.0	303.3	400.0	595.5
**9**	700.0	1003.3	1400.0	1995.5

Interval between doses was 15 minutes, with a total time of 2 hours and 15 minutes. Observation before the full therapeutic dose is 30 mins. IV cephalosporins used in this protocol included cefazolin, ceftriaxone, and cefepime.

Table S2. Rapid intravenous vancomycin desensitization protocol [2].

Premedication was performed with diphenhydramine 50mg IV and hydrocortisone 100mg IV 15 minutes prior to protocol initiation and q6h after protocol initiation.

**Dose Number**	**Vancomycin Dilution**	**Vancomycin Concentration (mg/mL)**	**Vancomycin Dose (mg)**	**Cumulative dose (mg)**
**1**	1:10,000	0.0002	0.02	0.02
**2**	1:1,000	0.002	0.20	0.22
**3**	1:100	0.02	2.0	2.22
**4**	1:10	0.2	20	22.22
**5**	Standard	2.0	500	522.22

Vancomycin Infusion Rate:
Initiate infusion at 0.5 mL/min (30mL/hr) and increase by 0.5 mL/min q 5 minutes as patient tolerates, to a maximum rate of 5 mL/min (300 mL/hr). If the patient experiences pruritis, hypotension, rash, or dyspnea, stop the infusion and restart at the most recently tolerated rate.

After the completion of dose 5, the full therapeutic dose in standard dilution should be administered immediately over a 2-hour infusion. Can decrease rate if patient becomes symptomatic increase rate as the patient tolerates the infusion. May administer diphenhydramine 60 minutes prior to each therapeutic dose.

Rapid vancomycin desensitization can be completed in 4 hours if the patient tolerates infusion rates as above.

Table S3. Slow intravenous vancomycin desensitization protocol [2].

Day	Dose Number	Vancomycin Dilution	Vancomycin Concentration (mg/mL)	Vancomycin Dose (mg)	Cumulative dose (mg)
**1**	1	0.5 mg in 500 mL	0.001	0.5	0.5
**2**	2	5.0 mg in 500 mL	0.01	5.0	5.5
**3**	3	10 mg in 500 mL	0.02	10	15.5
**4**	4	50 mg in 500 mL	0.10	50	65.5
**5**	4	50 mg in 500 mL	0.10	50	105.5
**6**	5	100mg in 500 mL	0.2	100	205.5
**7***	6	100 mg in 250 mL x 2	0.4	200	405.5
**8**	7	150 mg in 250 mL x 2	0.6	300	705.5
**9**	8	250mg in 250 mL x 2	1.0	500	1205.5
**10**	9	500 mg in 250 mL x 2	2.0	1000	2205.5
**11**	9	500mg in 250 mL x 2	2.0	1000	3205.5
**12**	9	500mg in 250 mL x 2	2.0	1000	4205.5
**13**	10	1000mg in 250 mL	4.0	1000	5205.5

***Starting on day 7, multiple infusions per day should be given consecutively**

Vancomycin Infusion Rate:

Each dose must be infused over 5 hours. If the patient experiences pruritis, hypotension, rash, or dyspnea, stop the infusion and restart at the most recently tolerated rate.

On day 14, administer the full therapeutic vancomycin dose in the normal dilution of NS (0.9% NaCl) or D5W (dextrose 5% in water), at an infusion rate of 100 mL/hour. Can decrease rate if patient becomes symptomatic or increase rate as the patient tolerates the infusion. May administer diphenhydramine 60 minutes prior to each therapeutic dose.

Table S4. Daptomycin desensitization protocol [3].

Dose Number	Dose (general)	Example Target Dose = 500mg
**1**	Desired dose x 10^{-6}	0.0005mg
**2**	Desired dose x 10^{-5}	0.005mg
**3**	Desired dose x 10^{-4}	0.05mg
**4**	Desired dose x 10^{-3}	0.5mg
**5**	Desired dose x 10^{-2}	5mg
**6**	Desired dose x 10^{-1}	50mg
**7**	Desired dose (full strength)	500mg

Each dilution was administered over 15 minutes; Interval between doses was 30 minutes.

Table S5. Oral clindamycin desensitization protocol [4].

Dose Number	Clindamycin Concentration (mg/mL)	Amount (mL)	Dose (mg)	Cumulative dose (mg)
**1**	0.02	0.25	0.005	0.005
**2**	0.02	2.5	0.05	0.055
**3**	0.2	2.5	0.5	0.56
**4**	2.0	2.5	5.0	5.56
**5**	20.0	0.5	10.0	15.6
**6**	20.0	1.0	20.0	35.6
**7**	20.0	2.0	40.0	75.6
**8**	20.0	4.0	80.0	156.0
**9**	Oral Capsule	N/A	150.0	306.0
Interval between doses was 30 minutes, with a total time of 4.5 hours.				

Table S6. Oral clarithromycin desensitization protocol [5].

Dose Number	Clarithromycin Concentration (mg/mL)	Amount (mL)	Dose (mg)	Cumulative dose (mg)
**1**	0.05	0.1	0.005	0.0
**2**	0.05	0.2	0.01	0.0
**3**	0.05	0.4	0.02	0.0
**4**	0.05	1	0.05	0.1
**5**	0.05	2	0.1	0.2
**6**	0.05	4	0.2	0.4
**7**	0.5	0.8	0.4	0.8
**8**	0.5	1.6	0.8	1.6
**9**	0.5	3.2	1.6	3.2
**10**	0.5	6.4	3.2	6.4
**11**	5	1.2	6	12.4
**12**	5	2.4	12	24.4
**13**	5	4.8	24	48.4
**14**	50	1	50	98.4
**15**	50	2	100	198.4
**16**	50	4	200	398.4
**17**	50	8	400	798.4
**18**	50	10	500	1298.4
Interval between doses was 15 minutes, with a total time of 4.5 hours.				

Table S7. Intravenous tobramycin desensitization protocol [6].

Dose Number	Tobramycin Dose (mg)	Cumulative dose (mg)	Complications
**1**	0.001	0.001	-
**2**	0.002	0.003	-
**3**	0.004	0.007	-
**4**	0.008	0.015	-
**5**	0.016	0.031	-
**6**	0.032	0.063	-
**7**	0.064	0.127	-
**8**	0.128	0.255	-
**9**	0.256	0.511	Transient rash
**10**	0.512	1.023	Transient rash
**11**	1.000	2.023	-
**12**	2.000	4.023	-
**13**	4.000	8.023	-
**14**	8.000	16.023	-
**15**	16.000	32.023	-
**16**	32.000	64.023	-
**17**	16.000	80.023	-
**Interval between doses was 30 minutes, for a total of 8 hours.**			

Table S8. Rapid Oral Sulfamethoxazole-Trimethoprim (SMX-TMP) Desensitization Protocol [7].

Dose Number	Amount (mL)	SMX/TMP Dose (mg)	Cumulative Dose (mg)
**0**	0.1	4/0.8	4/0.8
**1**	0.2	8/1.6	12/2.4
**2**	0.4	16/3.2	28/5.6
**3**	0.8	32/6.4	60/12.0
**4**	1.6	64/12.8	124/24.8
**5**	3.2	128/25.6	252/50.4
**6**	6.4	256/51.2	508/101.6
**7**	10	400/80	908/181.6
Interval between doses was one hour, with a total time of 7 hours.			

Table S9. Slow Oral Sulfamethoxazole-Trimethoprim (SMX-TMP) Desensitization Protocol [7].

Day	SMX/TMP Doses	Day	SMX/TMP Doses
**1**	Dose 1: 0.02 μg/0.004 μg<br>Doses 2-4: Increase dose stepwise by 0.02 μg/0.004 μg.	**6**	Dose 1: 2 mg/400 μg<br>Doses 2-4: Increase dose stepwise by 2 mg/400 μg.
**2**	Dose 1: 0.2 μg/0.04 μg<br>Doses 2-4: Increase dose stepwise by 0.2 μg/0.04 μg.	**7**	Dose 1: 20 mg/4 mg<br>Doses 2-4: Increase dose stepwise by 20 mg/4 mg.
**3**	Dose 1: 2 μg/0.4 μg<br>Doses 2-4: Increase dose stepwise by 2 μg/0.4 μg.	**8**	Dose 1: 40 mg/8 mg<br>Dose 2: 80 mg/16 mg<br>Dose 3: 160 mg/32 mg<br>Dose 4: 320 mg/64 mg
**4**	Dose 1: 20 μg/4 μg<br>Doses 2-4: Increase dose stepwise by 20 μg/4 μg.	**9**	Dose 1: 200 mg/40 mg<br>Dose 2: 200 mg/40 mg<br>Dose 3: 400 mg/80 mg
**5**	Dose 1: 0.2 mg/40 μg<br>Doses 2-4: Increase dose stepwise by 0.2 mg/40 μg.	**10**	Dose 1: 800 mg/160 mg<br>Dose 2: 800 mg/160 mg

Interval between doses in days 1-9 was 30 minutes. Interval between therapeutic doses on day 10 was 3 hours.

Table S10. Intravenous metronidazole desensitization protocol [8].

Step	Dose (mg)	Concentration	Volume (mL)
**1**	0.005	0.005 mg/mL	1.0
**2**	0.015	0.005 mg/mL	3.0
**3**	0.05	0.05 mg/mL	1.0
**4**	0.15	0.05 mg/mL	3.0
**5**	0.5	0.5 mg/mL	1.0
**6**	1.5	0.5 mg/mL	3.0
**7**	5	5.0 mg/mL	1.0
**8**	15	5.0 mg/mL	3.0
**9**	30	5.0 mg/mL	6.0
**10**	60	5.0 mg/mL	12.0
**11**	125	5.0 mg/mL	25.0
**12**	250	250 mg orally	Tablet
**13**	500	500 mg orally	Tablet
**14**	2000	2000 mg orally	Tablet

Intravenous doses should be administered every 15-20 minutes, whereas oral doses should be administered at 1-hour intervals.

Table S11. Oral metronidazole desensitization protocol [8].

Step	Dose (mg)	Concentration	Volume (mL)
**1**	0.00025	0.025 mg/mL	1.0
**2**	0.025	0.025 mg/mL	3.0
**3**	0.25	0.25 mg/mL	1.0
**4**	2.5	2.5 mg/mL	3.0
**5**	25	2.5 mg/mL	1.0
**6**	250	250 mg	Tablet
**7**	750	750 mg	Tablet
**8**	1000	1000 mg	Tablet

Doses should be administered over a 24-hour period.

Table S12. Intravenous ciprofloxacin desensitization protocol [9].

Step	Concentration (mg/mL)	Volume (mL)	Amount administered (mg)	Cumulative total dose (mg)
**1**	0.1	0.1	0.01	0.01
**2**	0.1	0.2	0.02	0.03
**3**	0.1	0.4	0.04	0.07
**4**	0.1	0.8	0.08	0.15
**5**	1	0.16	0.16	0.32
**6**	1	0.32	0.32	0.63
**7**	1	0.64	0.64	1.27
**8**	2	0.6	1.2	2.47
**9**	2	1.2	2.4	4.87
**10**	2	2.4	4.8	9.67
**11**	2	5	10	19.67
**12**	2	10	20	39.67
**13**	2	20	40	79.67
**14**	2	40	80	159.67
**15**	2	120	248	399.67

Doses should be administered in 15-minute interval over a 4-hour period. Once complete, the therapeutic regimen (400 mg IV every 12 hours) should begin 4 hours later. Between therapeutic doses, 25 mg IV supplemental doses should be administered.

Table S13. Intravenous liposomal amphotericin B (LAmB) desensitization protocol [10].

Step	Concentration (mg/mL)	Volume (mL)	Rate (mL/hr)	Amount administered (mg)	Cumulative total dose (mg)
**1**	0.00004	10	40	0.0004	0.0004
**2**	0.0004	10	40	0.004	0.0044
**3**	0.004	10	5	0.04	0.0444
**4**	0.04	10	5	0.4	0.4444
**5**	0.4	10	5	4	4.4444
**6**	0.66	60	30	40	44.4444
**7**	1.4	280	140	400	444.4444

Doses should be administered in 2-hour intervals.

Table S14. Rapid oral fluconazole desensitization protocol [11].

Step	Concentration (mg/mL)	Volume (mL)	Amount administered (mg)	Cumulative total dose (mg)
**1**	0.02	1	0.02	0.02
**2**	0.02	2	0.04	0.06
**3**	0.02	4	0.08	0.14
**4**	0.2	0.6	0.16	0.3
**5**	0.2	1.6	0.32	0.62
**6**	0.2	3.2	0.64	1.26
**7**	2	0.75	1.5	2.76
**8**	2	1.5	3	5.76
**9**	2	3	6	11.76
**10**	20	0.6	12	23.76
**11**	20	1.2	24	47.76
**12**	20	2.5	50	97.76
**13**	20	5	100	197.76

Doses should be administered in 15-minute intervals.

Table S15. Several-day oral fluconazole desensitization protocol [12].

Step	Concentration (mg/mL)	Volume (mL)	Amount administered (mg)
**1**	2	100 µL	200 µg
**2**	2	1 mL	2 mg
**3**	2	2 mL	4 mg
**4**	2	4 mL	8 mg
**5**	2	8 mL	16 mg
**6**	2	15 mL	30 mg
**7**	2	30 mL	60 mg
**8**	2	60 mL	120 mg
**9**	---	Tablet	200 mg
**10**	---	Tablet	200 mg

Doses should be administered in 6-hour intervals.
Premedication with oral diphenhydramine 25 mg and famotidine 20 mg should be administered 30 minutes prior to protocol initiation.
Oral diphenhydramine 25 mg three times per day and famotidine 20 mg twice per day should be continued throughout the protocol.

Table S16. Oral itraconazole capsule desensitization protocol [13].

Step	Amount administered (mg)
**1**	1
**2**	2
**3**	4
**4**	8
**5**	16
**6**	32
**7**	64
**8**	128
**9**	200

Doses should be administered in 30-minute intervals.
Oral capsules were crushed. The contents were then weighed and mixed in applesauce for administration.

29 **Table S17.** Intravenous voriconazole desensitization protocol [11].

Step	Concentration (mg/mL)	Amount administered (mg)	Cumulative total dose (mg)
**1**	0.1	0.02	0.02
**2**	0.1	0.05	0.07
**3**	0.1	0.1	0.17
**4**	1	0.25	0.42
**5**	1	0.5	0.92
**6**	1	1	1.92
**7**	5	2	3.92
**8**	5	4	7.92
**9**	5	8	15.92
**10**	5	16	31.92
**11**	5	32	63.92
**12**	5	64	127.92
**13**	5	128	255.92[a]
**14**	5	207	335[b,c]

Doses should be administered in 15-minute intervals.
[a] Equivalent to 4 mg/kg maintenance dose in this patient
[b] Equivalent to 6 mg/kg loading dose in this patient
[c] After completion of the protocol, a second loading dose was administered followed by maintenance doses every 12 hours.

30 **Table S18.** Oral acyclovir desensitization protocol [14].

Step	Dose (mg)	Concentration (mg/ml)	Volume (mL)
***1**	0.04	0.4	0.1
**2**	0.1	0.4	0.25
**3**	0.2	0.4	0.5
**4**	0.4	0.4	1.0
**5**	0.8	4	0.2
**6**	1.6	4	0.4
**7**	3.2	4	0.8
**8**	6	4	1.5
**9**	12	40	0.3
**10**	24	40	0.6
**11**	50	40	1.25
**12**	100	40	2.5
**13**	200 mg oral tablet	N/A	N/A
**14**	400 mg oral tablet	N/A	N/A
**15**	800mg oral tablet	N/A	N/A

**40mg of oral prednisolone was administered 60 minutes prior to starting step one of this protocol*
Interval between doses was 15 minutes
N/A: not applicable.

31

Table S19. Oral valganciclovir desensitization protocol [15].

Step	Dose (mg)	Total Dose (mg)
**1**	0.1	0.1
**2**	0.2	0.3
**3**	0.4	0.7
**4**	0.8	1.5
**5**	1.6	3.1
**6**	3.5	6.6
**7**	7	13.6
**8**	14	27.6
**9**	28	55.6
**10**	58	113.6
**11**	115	228.6
**12***	225	453.6

Interval between doses was 15 minutes.
**After step 12, next doses were 8-12 hours later with a 450mg oral tablet, followed by 450mg orally twice daily.*

References:

1. Win, P.H.; Brown, H.; Zankar, A.; Ballas, Z.K.; Hussain, I. Rapid intravenous cephalosporin desensitization. *J Allergy Clin Immunol* **2005**, *116*, 225-228, doi:10.1016/j.jaci.2005.03.037.
2. Wazny, L.D.; Daghigh, B. Desensitization protocols for vancomycin hypersensitivity. *Ann Pharmacother* **2001**, *35*, 1458-1464, doi:10.1345/aph.1A002.
3. Metz, G.M.; Thyagarajan, A. A successful protocol for daptomycin desensitization. *Ann Allergy Asthma Immunol* **2008**, *100*, 87, doi:10.1016/S1081-1206(10)60411-5.
4. Esty, B.; Minnicozzi, S.; Chu, E.C.; Broyles, A.D.; Yee, C.S.K. Successful rapid oral clindamycin desensitization in a pediatric patient. *J Allergy Clin Immunol Pract* **2018**, *6*, 2141-2142, doi:10.1016/j.jaip.2018.04.004.
5. Holmes, N.E.; Hodgkinson, M.; Dendle, C.; Korman, T.M. Report of oral clarithromycin desensitization. *Br J Clin Pharmacol* **2008**, *66*, 323-324, doi:10.1111/j.1365-2125.2008.03192.x.
6. Earl, H.S.; Sullivan, T.J. Acute desensitization of a patient with cystic fibrosis allergic to both beta-lactam and aminoglycoside antibiotics. *J Allergy Clin Immunol* **1987**, *79*, 477-483.
7. Patriarca, G.; Schiavino, D.; Buonomo, A.; Aruanno, A.; Altomonte, G.; Nucera, E. Desensitization to co-trimoxazole in a patient with fixed drug eruption. *J Investig Allergol Clin Immunol* **2008**, *18*, 309-311.
8. Helms, D.J.; Mosure, D.J.; Secor, W.E.; Workowski, K.A. Management of trichomonas vaginalis in women with suspected metronidazole hypersensitivity. *Am J Obstet Gynecol* **2008**, *198*, 370 e371-377, doi:10.1016/j.ajog.2007.10.795.
9. Gea-Banacloche, J.C.; Metcalfe, D.D. Ciprofloxacin desensitization. *J Allergy Clin Immunol* **1996**, *97*, 1426-1427.
10. Shadur, B.; Trahair, T.N.; O'Brien, T.; Russell, S.J.; Ziegler, J.B. Desensitisation to liposomal amphotericin B. *J Allergy Clin Immunol Pract* **2017**, *5*, 181-183, doi:10.1016/j.jaip.2016.08.006.
11. Randolph, C.; Kaplan, C.; Fraser, B. Rapid desensitization to fluconazole (Diflucan). *Ann Allergy Asthma Immunol* **2008**, *100*, 616-617, doi:10.1016/S1081-1206(10)60063-4.
12. Jariwala, S.; Vernon, N.; de Vos, G. A novel method of desensitization for fluconazole hypersensitivity in a patient with AIDS. *Ann Allergy Asthma Immunol* **2011**, *106*, 542-543, doi:10.1016/j.anai.2011.02.020.
13. Bittleman, D.B.; Stapleton, J.; Casale, T.B. Report of successful desensitization to itraconazole. *J Allergy Clin Immunol* **1994**, *94*, 270-271.
14. Snape, S.E.; Finch, R.G.; Venkatesan, P. Aciclovir desensitisation and rechallenge. *BMJ Case Rep* **2011**, *2011*, doi:10.1136/bcr.10.2010.3392.
15. Gonzalez-Estrada, A.; Fernandez, J. Novel valganciclovir desensitization protocol. *Transplantation* **2014**, *98*, e50-51, doi:10.1097/TP.0000000000000320.
